# Nonurgent Visits to the Pediatric Emergency Department before and during the First Peak of the COVID-19 Pandemic

**DOI:** 10.1155/2022/7580546

**Published:** 2022-02-28

**Authors:** Laura Guckert, Heiko Reutter, Nadia Saleh, Rainer Ganschow, Andreas Müller, Fabian Ebach

**Affiliations:** ^1^Dept. of Pediatrics, Children's Hospital, University Hospital Bonn, Venusberg-Campus 1, Gebäude 33 53127 Bonn, Germany; ^2^Div. of Neonatology and Pediatric Intensive Care Medicine, Dept. of Pediatric and Adolescent Medicine, Friedrich-Alexander-University Erlangen-Nürnberg, Erlangen, Germany; ^3^Dept. of Neonatology and Pediatric Intensive Care, Children's Hospital, University Hospital Bonn, Germany

## Abstract

**Background:**

Nonurgent visits in pediatric Emergency Departments are a growing burden. In order to find predictors for those nonurgent visits, we performed a retrospective analysis of unscheduled visits at the Pediatric Emergency Department of the University Hospital of Bonn, Germany, in the year 2017. Additionally, we compared these findings to unscheduled visits during the first peak of the worldwide pandemic of the Coronavirus disease 2019, to see if there would be an effect on nonurgent pediatric Emergency Department attendances.

**Methods:**

For our retrospective cohort study, we analyzed more than 5.000 visits at the pediatric Emergency Department of the University Hospital of Bonn, Germany, before and during the first peak of the ongoing worldwide pandemic of the Coronavirus disease 2019, particularly with regard to their urgency. Data included gender, age, zip code, urgency, and preexisting conditions.

**Results:**

Our study shows that more than half of unscheduled pediatric Emergency Department visits (69%) at the University Hospital in Bonn are for nonurgent reasons, with short living distance being a factor to present children to a pediatric Emergency Department, even with minor complaints. During the first peak of the pandemic of the Coronavirus disease 2019, nonurgent visits decreased significantly, potentially due to hesitation to attend a pediatric Emergency Department with minor issues, fearing an infection with SARS-CoV-2 at the hospital.

**Conclusion:**

Many people use pediatric Emergency Departments for nonemergency complaints. In order to address the reasons for nonurgent visits to pediatric Emergency Departments and to prevent parents from doing so, further studies and targeted education concepts for parents are needed.

## 1. Introduction

Emergency Department (ED) overcrowding is a growing phenomenon in many countries worldwide, as a range of studies show [1–4]. Although there is limited pediatric-specific data available, there is evidence that overcrowding can also be observed in pediatric EDs [5–10].

Despite a broad net of registered general practitioners and pediatricians in first world countries, it was noted in some studies that people often rather visit EDs to seek for medical help due to many different reasons [11–13]. Consequences of the resulting overcrowding include longer waiting times [10, 11], rising costs for the health care system [14], short time slots for individual patients, and the need for multitasking and frequent interruptions [15]. Especially, these frequent and often recurrent interruptions delay treatment for patients in need [3, 16] and decrease the quality of diagnosis and therapy [17].

Although being generally described as an emerging problem, numbers of nonurgent visits at pediatric EDs differ between countries, ranging from 40% in a Belgian study [12] to 79% in a study from Italy [6]. Studies from Argentina and Germany [18, 19] report nonurgent visits to account for 59% and 53% of all pediatric ED presentations, respectively.

Additionally, a Canadian study found that between 2002 and 2011, the number of nonurgent visits to pediatric EDs in Canada is not only high but increasing both in absolute numbers and in relation to all pediatric ED visits [7].

Some studies suggest that the availability of EDs in close proximity is one of the main predictors for visiting a pediatric ED in nonurgent cases [12, 19], with other reasons including long waiting times for an appointment in a primary care center [8, 18], lack of availability of a Primary Care Physician (PCP), need for medical attention outside PCPs' working hours [19, 20], and the assumption of a greater amount of diagnostic resources in a pediatric ED [13, 19].

In addition, socioeconomic characteristics [10], overestimation of urgency, wrong assessment of disease severity, and the need for immediate medical reassurance [11, 21–23] were found to be motivators for nonurgent visits at a pediatric ED.

The aim of our study was to identify predictors for nonurgent visits to the pediatric ED and evaluate if the beginnings of the pandemic of the Coronavirus disease 2019 (COVID-19 pandemic) had an influence on those visits.

## 2. Methods

### 2.1. Data Sources

Every year, the pediatric ED of the University Children's Hospital Bonn attends to approximately 7,000 emergency visits (by a total number of 35,000 emergency attendances at all sections of the University Hospital).

For our retrospective cohort study, we surveyed the electronic database of the pediatric ED of the University Hospital of Bonn in the year 2017 as well as during the first peak of the COVID-19 pandemic in March and April of 2020, in order to explore predictors for nonurgent pediatric ED visits.

### 2.2. Selection Criteria and Search Strategy

Gender, age, date of visit, zip code, assessment of urgency, preexisting medical conditions, and final diagnosis made at the pediatric ED were extracted from the electronic data files of the year 2017 and of March and April 2020. Neither outcomes nor admission rates were analyzed.

Since in Germany adolescents tend to consult general practitioners rather than pediatricians and therefore could be underrepresented, we excluded patients older than 16 years from our study. This is in accordance with some previous studies [8, 24], thus allowing comparability.

**Figure 1:**
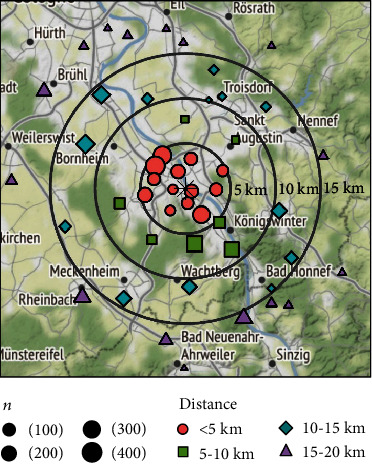
Number of patients per zip code area and distance to pediatric ED (∗) in the year 2017 (Map tiles by Stamen Design, under CC BY 3.0. Data by OpenStreetMap, under ODbL).

Using the registered zip codes of the patients' home address, we calculated the geographical distance between the zip code area's geographical center and our pediatric ED for each patient (Figure 1). As Bonn is located in a very populous part of Germany, with a wide coverage of hospitals and EDs, patients living further away than 20 km (and therefore in all likelihood closer to another pediatric ED than ours) were excluded from our study.

Urgency was assessed upon presentation by trained pediatric ED nurses following the Manchester Triage System (MTS) algorithms [25], used in a modified version adjusted to the German-speaking part of the world. The resulting categories are represented by the colors red, orange, yellow, green, and blue, coding for the maximum time to the first contact with a physician (red = immediately, orange = 10 minutes, yellow = 30 minutes, green = 90 minutes, and blue = 120 minutes) [26].

In our study, categories blue and green were classified as nonurgent visits, and categories yellow to red were classified as urgent visits.

Patients' final diagnoses were grouped into six categories: respiratory, abdominal, skin, trauma, neurological, and other.

### 2.3. Statistical Analysis

Due to the nonnormal distribution of our data, medians were calculated and compared using the Mann–Whitney *U* test. In order to determine factors leading to a nonurgent presentation at the ED, crude odds ratios (ORs) were calculated. Additionally, adjusted ORs were computed using logistic regression through the backwards method, correcting for gender, age, distance to the ED, out of hours visit, and preexisting medical conditions. All tests were two-tailed on a 5% significance level with the post hoc Bonferroni correction, if applicable. Data was analyzed using the R statistical environment [27].

## 3. Results

Of all 6,254 recorded visits in both analyzed time frames, a total of 5,410 met the inclusion criteria (age 0-16 years, geographical distance between 0 and 20 km). From January 2017 to December 2017, 5,038 unscheduled visits by 4,115 patients (54.6% male, 45.3% female) were included (Table 1). 11% (*n* = 442) had a preexisting medical condition of any kind, accounting for 13% (*n* = 631) of pediatric ED presentations. Visits were classified as “urgent” in 1,582 cases (31%), including 1,288 yellow, 239 orange, and 55 red cases according to the MTS. All other cases were classified as “nonurgent” (*n* = 3,456; 69%).

**Figure 2:**
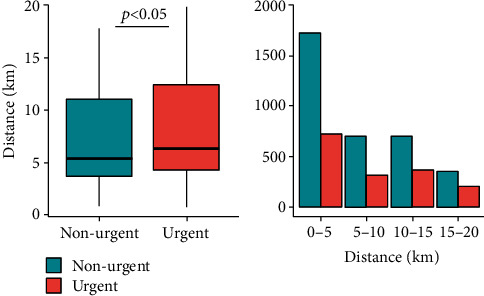
Linear distance to the pediatric ED for urgent and nonurgent visits in 2017.

**Figure 3:**
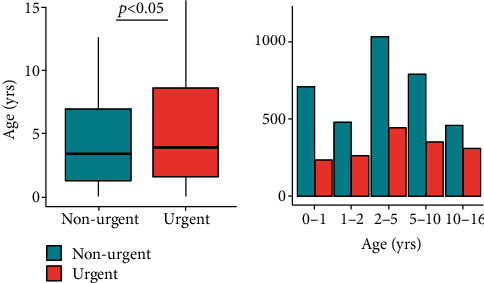
Age distribution in urgent and nonurgent visits in 2017.

**Figure 4:**
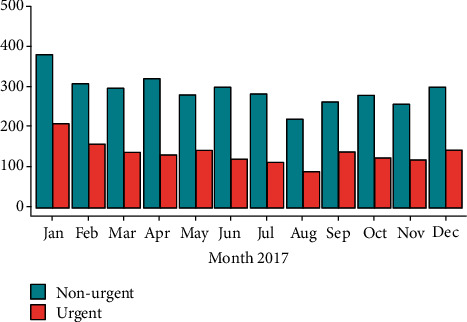
Seasonal comparison between urgent and nonurgent visits in 2017.

**Figure 5:**
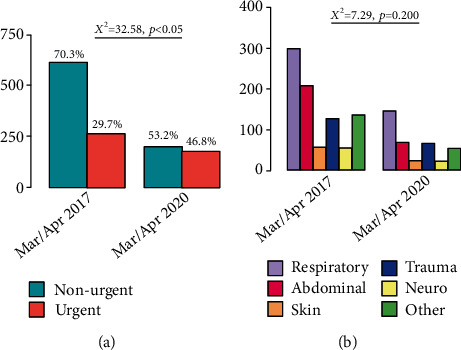
(a) Nonurgent and urgent visits to the pediatric ED and (b) preliminary diagnoses in March and April 2017, respectively, compared to March and April 2020 (first peak of COVID-19 pandemic).

Patients' median age was 3.54 years (IQR 1.41 y–7.45 y), and median distance to the hospital was 5.4 km (IQR 3.7 km–12.3 km). Median linear distance to the pediatric ED for children with urgent presentations was 6.2 km (IQR 4.2 km–12.3 km). For children with nonurgent presentations, median distance to the pediatric ED was 5.4 km (IQR 3.7 km–10.9 km) (*p* < 0.05) (Figure 2). Children, whose presentation was classified as urgent, were older (median 3.9 years, IQR 1.6-8.6 years) than children who were presented for nonurgent complaints (median 3.4 years, IQR 1.3-7.0 years) (*p* < 0.05) (Figure 3). The main category of symptoms leading to presentation was respiratory (*n* = 1,659, 33%), followed by abdominal (*n* = 1,002, 20%), other (*n* = 894, 18%), trauma (*n* = 881, 17%), skin (*n* = 357, 7%), and neurological (*n* = 245, 5%). In a multivariate analysis, absence of preexisting medical condition, age < 2y, distance to the pediatric ED < 5 km, and a visit within office hours were found to be predictors for a nonurgent presentation. In spite of well-known seasonal patterns for different diseases, with a peak of respiratory infections in winter months in temperate climate being the most prominent example [28], we could not find significant differences regarding the urgency of presentation between summer and winter (*χ*^2^ = 2.94, *p* = 0.086) (Figure 4). During the first peak of the COVID-19 pandemic in Germany in March and April of 2020, we additionally analyzed 372 visits by 355 patients (56.6% male, 43.4% female). The total number of visits decreased by 57% compared to March and April of 2017, with a decrease of nonurgent visits of 68% (Figure 5(a)). The number of urgent visits only decreased by 33% (*χ*^2^ = 32.6, *p* < 0.05). Grouped diagnoses at presentation did not differ significantly between March/April of 2017 and March/April of 2020. (*χ*^2^ = 7.3, *p* = 0.2) (Figure 5(b)).

## 4. Discussion

By using the administrative database of the University Hospital of Bonn, we could analyze a large number of records of pediatric ED visits of the year 2017 and of March and April of 2020, during the first peak of the COVID-19 pandemic. We found that at the University Hospital of Bonn, the majority of unscheduled visits in the pediatric ED is for nonurgent reasons. This is mostly in line with previous retrospective findings from different European and international groups [4, 6, 12, 18, 29].

The statutory health insurance system in Germany, which is structured into ambulatory/outpatient care, hospitals, and ambulant as well as inpatient rehab facilities, provides free health care for all citizens. This is financed through mandatory insurance fees paid by every employee and entrepreneur in Germany. Therefore, visits in pediatric EDs and appointments at PCPs are both free of charge. Patients have a free choice of medical practitioners. Considering this system, economic factors are unlikely to play a major role in parents' decisions to seek medical advice, compared to, e.g., the United States, where the decision to visit a pediatric ED often is influenced by the individual health insurance coverage [30].

We could confirm short linear distance from home to the hospital as a predictor for nonurgent presentations in our analysis. Previous retrospective analyses in Italy showed short distance to be an influencing factor for inappropriate pediatric ED use [24]. A study carried out in Australia found that residing close to the pediatric ED is a factor associated with increased nonurgent pediatric ED presentations [31]. These findings underline results of questionnaire-based approaches in Belgium, which also show distance to an ED to be significantly associated with inappropriate ED use [12]. Surprisingly, presentation to the pediatric ED within office hours was also found as a predictor for nonurgent visits. This could be explained by parents, who recognize the nonurgent nature of their children's complaints and refrain from visiting the pediatric ED at nighttime, but nevertheless prefer the ED to a PCP's office for different possible reasons. For example, due to difficulties to obtain an appointment at the PCP as close in time as needed, parents assume longer waiting times at the PCP and the assumption to get a faster treatment and a higher quality of diagnostics at a pediatric ED.

Furthermore, the absence of preexisting medical condition was found to be a predictor for nonurgent reasons to attend a pediatric ED. This could be expected, as it is very likely that children with preexisting medical conditions usually have a higher risk of severe problems and therefore are, more than others, classified as urgent visits (e.g., a child with known epilepsy being presented with a convulsion). We also found age < 2 years to be a predictor for nonurgent complaints being presented at a pediatric ED, as it is very likely that parents are often alarmed faster during their child's first two years of life, due to many different reasons (difficulties of communication with a baby/toddler, misinterpretation of their symptoms, needs and problems, and higher risk of infections due to kindergarten). This goes in line with previous findings [6, 10]. During the first lockdown period in Germany, caused by the worldwide COVID-19 pandemic, everyday life was subject to extensive restrictions. Schools and kindergartens were closed; working in home office was strongly encouraged; social contacts were strictly limited; cafés, restaurants, and retail shops were closed; and all cultural and leisure time activities were prohibited, as they did not ensure proper social distancing.

Considering the decrease in visits of 57% during the first peak of the COVID-19 pandemic in Germany in March and April 2020, with a decrease of nonurgent visits of 68% compared to March and April 2017, we assume that, most likely, parents were hesitant to present their children for minor issues, fearing they could be contracting the virus at the hospital. This presumption goes along with data from other studies conducted in Europe and abroad, showing evidence of the hesitant use of pediatric ED facilities for nonurgent reasons during the COVID-19 pandemic [32–35]. Although the restrictions concomitant with the lockdown period could have led, due to limited social contacts and therefore less transmission of infections, to a decrease in, e.g., gastroenteritis or common cold, our study shows that grouped diagnoses did not differ significantly compared to those in March and April of 2017.

Given that overcrowding due to nonurgent visits is an increasing challenge for pediatric EDs [6, 7], there have been numerous attempts to reduce the number of nonurgent visits by educational measures [36, 37]. A British randomized, prospective cohort study compared two groups of parents to assess the impact of a short educational video, shown to them during an ED visit for minor complaints [37]. Even though knowledge and assessment of childhood fever improved, ED use for minor complaints did not decrease. On the other hand, a study carried out in Texas, United States, evaluating educational measures for parents of children with asthma, showed that parental education is related with a better understanding and situational evaluation of the disease and boosts self-confidence in handling asthmatic spells [36]. Although this study does not distinguish between urgent and nonurgent cases, the number of times children with asthma was presented in the pediatric ED and was significantly reduced after these educational measures. Even though the results of educational measures remain ambiguous, improving their knowledge and ability could have reassuring effects on parents, thus enabling them to handle nonurgent complaints at home.

## 5. Conclusion

In conclusion, our study shows that more than half (69%) of unscheduled pediatric ED visits at the University Hospital in Bonn are for nonurgent reasons, with short living distance, younger age, attendance within office hours, and absence of preexisting medical condition being predictors for a nonurgent presentation. During the first peak of the COVID-19 pandemic in Germany, nonurgent visits decreased significantly compared to the respective months of 2017, potentially due to parents' hesitation to attend a pediatric ED with minor issues, fearing their children contracting SARS-CoV-2 at the hospital. Although we can assume that parents' decision for unscheduled pediatric ED visits are intrinsically related to availability, capacity, and quality of primary care, more studies are needed, to further explore motives and standpoints having an effect on parents' decision. Strategies and solutions to avoid the inappropriate use of pediatric EDs should be implemented and thereby reduce worldwide pediatric ED overcrowding. Especially interviews with parents/families living in short living distance to pediatric EDs would be helpful and interesting.

## 6. Study Limitations

As this is a retrospective study with analyzed data of more than 5,000 visits of patients in our pediatric ED, we did not conduct follow-up interviews with the parents and, therefore, can only speculate about individual motives to use a pediatric ED for nonurgent reasons. We only analyzed the first lockdown period of the COVID-19 pandemic, resulting in a small sample size compared to the data collection of 2017. Unfortunately, additional information, e.g., aspects of nationality and culture, language, socioeconomic status, or educational background of families such as patients being an only child or having siblings, was not possible to obtain retrospectively and therefore could not be analyzed. We did not conduct surveys of the availability of PCPs, their waiting times, and opening hours. In retrospect, this might have given us information of potentially controllable factors of inappropriate presentations at the pediatric ED. As there are two more pediatric EDs within the analyzed radius of 20 km, children living in close proximity to these facilities could be underrepresented in our study. Although designed to maximize interrater reliability, performance of the MTS is always subject to the individual nurse's training and experience [38].

## Figures and Tables

**Table 1:** Distribution of patients including age, gender, assessment of urgency, and distance to the pediatric ED in 2017.

	Nonurgent *N* = 3456	Urgent *N* = 1582
Gender:		
Male	1905 (55.1%)	874 (55.2%)
Female	1551 (44.9%)	708 (44.8%)
Age group:		
<1 y	705 (20.4%)	230 (14.5%)
1 y to <2 y	477 (13.8%)	257 (16.2%)
2 y to <5 y	1029 (29.8%)	442 (27.9%)
5 y to <10 y	789 (22.8%)	348 (22.0%)
>10 y	456 (13.2%)	305 (19.3%)
Distance:		
<5 km	1727 (50.0%)	718 (45.4%)
5 km–10 km	690 (20.0%)	306 (19.3%)
10 km–15 km	700 (20.3%)	363 (22.9%)
>15 km	339 (9.8%)	196 (12.4%)
Out of office hoursª:		
No	1224 (35.4%)	458 (29.0%)
Yes	2232 (64.6%)	1124 (71.0%)
Medical condition^b^:		
No preexisting condition	3073 (88.9%)	1334 (84.3%)
Preexisting condition	383 (11.1%)	248 (15.7%)

ªVisits to pediatric EDs after PCP's office hours. ^b^These patients suffered from a preexisting medical condition.

## Data Availability

All data are available upon request.

## Abbreviations

ED: Emergency Department
COVID-19: Coronavirus disease 2019
PCP: Primary Care Physician
MTS: Manchester Triage System
ORs: Odds ratios
SARS-CoV-2: Severe acute respiratory syndrome Coronavirus type 2.

## Additional Points

*Reporting Checklist*. The authors have completed the STROBE reporting checklist.
